# The Effect of SARS-COV‑2 Protein Fragments on the Dimerization of α‑Synuclein

**DOI:** 10.1021/acschemneuro.5c00635

**Published:** 2025-10-22

**Authors:** Lucy M. Coleman, Ulrich H. E. Hansmann

**Affiliations:** Department of Chemistry & Biochemistry, 6187 University of Oklahoma, Norman, Oklahoma 73019, United States

**Keywords:** SARS-CoV-2, α-synuclein, dimers, amyloid aggregation, Parkinson’s disease, molecular dynamics simulations

## Abstract

There is evidence that amyloidogenic segments in SARS-COV-2 proteins can induce aggregation of α-synuclein (αS), the main component of brain-located amyloids whose presence is connected with Parkinson’s Disease (PD). Using molecular dynamics simulations, we showed in earlier work that SARS-COV-2 protein fragments shift the ensemble of αS chains toward more aggregation-prone conformations. However, the mechanism by which these chains assemble into fibrils, the presumed neurotoxic agents in PD, is not clear. The first step on that route is the formation of dimers. For this reason, we have now, using again molecular dynamics simulations, studied how the fragment _194_FKNIDGYFKI_203_ (FI10) of the SARS-COV-2 spike protein and the fragment _54_SFYVYSRVK_62_ (SK9) of the envelope protein alter the ensemble of α-synuclein dimers. Our simulations suggest a differential stabilization of such dimers that would preferentially seed rod-like fibrils over the competing twister-like structures.

## Graphical abstract

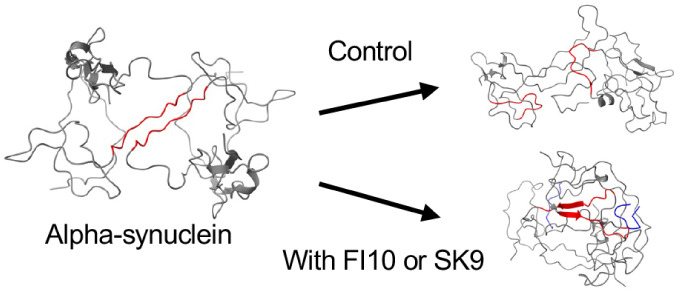

## 1 Introduction

A hallmark of Parkinson’s disease (PD) is the presence of amyloids located in the brains of patients that are made mainly of α-synuclein (αS) and appear to be the neurotoxic agent.^1,2^ As correlations have been observed between falling ill with COVID-19 and outbreaks of PD,^3^ and SARS-COV-2-induced αS amyloid formation has been found *in vitro,* ^4^ we and other groups^5^ have proposed that amyloidogenic SARS-COV-2 protein regions can enhance the aggregation of αS, potentially causing PD. We have speculated that during acute inflammation, as commonly seen in COVID-19, neutrophils release enzymes that cleave SARS-COV-2 proteins into amyloidogenic fragments, which in turn cross-seed human proteins. Such cleavage has been shown for the amyloidogenic segment of residues _194_FKNIDGYFKI_203_ (FI10) of the spike protein.^6^ Using all-atom molecular dynamics simulations, we have shown that this peptide and the segment _54_SFYVYSRVK_62_ (SK9) of the envelope protein shift the ensemble of αS monomers toward conformations that are more aggregation prone.^7,8^ However, the likely neurotoxic agents in PD are not these monomers but their assemblies into fibrils that are characterized by a cross-beta structure; and the pathogenesis and severity of PD are correlated with the structures of the polymorphic fibrils in the patients’ brains.

The first step on the road to such fibrils is dimers that may serve as their seeds. This is the reason why we investigate, in the present study, the effect of SARS-COV-2 protein fragments on the stability of the dimers. For this purpose, we employ all-atom molecular dynamics simulations, where the above two SARS-COV-2 protein fragments interact with αS dimers built from aggregation-prone monomer configurations collected in our earlier work. We are especially interested to see if any stabilizing effect would depend also on the αS dimer structure, and therefore also leading to specific fibril forms, or if the earlier observed differential stabilization of fibril polymorphs^8^ depends only on the energetics of the fibril conformations but not on the kinetics of their formation.

## 2 Results and Discussion

In previous work, we could show that interaction with SARS-COV-2 protein fragments such as SK9 or FI10 shifts the ensemble of αS chains toward more extended and aggregation-prone conformations. We have argued that the observed distribution would favor the formation of rod-like fibrils, as the presence of the two viral protein fragments leads to an increased exposure of residues E46–A56, i.e., the segment that forms the binding interface of the protofibrils in the rod fibril polymorph. Reanalyzing our old data, we find for this segment in the simulations an average root-mean-square deviation (RMSD) to the corresponding chain segment in the rod fibril of about 5 Å for the control, but only 3 Å in the presence of SK9, and 4 Å in the presence of FI10. On the other hand, for the segment G68-A78 where in twister fibrils the protofibrils pack, the RMSD is about 4 Å, similar in the control and in the presence of SK9 or FI10. Note that these averages have to be taken with a grain of salt, as they are derived from all trajectories of refs. 7 and 8, which differ in length and number of trajectories. However, even when taking these limitations into account, these averages show that rod-like or twister-like regions do appear in the ensemble of monomer conformations, and their frequency may depend on the presence or absence of the viral protein fragments.

Our assumption is that dimers may form by the binding of two αS chains at these two segments, leading to dimers that later would seed the formation of either rod-like or twister-like αS. Assuming a threshold of 3.5 Å, we find that in the control simulation about 20% of conformations have rod-like segments, while in the presence of SK9 or FI10, the frequency increases to about 50%. On the other hand, twister-like segments are seen in about 50% of the conformations in the control simulation or in the presence of SK9, but only in about 40% of the conformations in simulations with FI10 present. These frequencies suggest that in the presence of the viral protein fragments, there is a higher probability of forming dimers by binding at segment E46-A56 than seen in the control but not for forming dimers that bind at segment G68-A78. This is interesting because binding at the rod-binding interface may lead to dimers that could serve as templates for the attachment of further chains, thereby increasing the probability that subsequent oligomer species resemble rod-like protofibrils and can elongate into mature rod-like fibrils. Meanwhile, the lower frequencies for twister-like segments seem to make a similar pathway less likely for the competing twister-like fibrils. Albeit our monomer simulations suggest that the presence of viral protein fragments increases the probability of aggregates binding at the same segment as in rod-like fibrils, we ignored this propensity difference in the monomer distributions (already reported by us in earlier work^8^) when setting up our dimer simulations. In order to avoid any bias, we instead generated model dimer configurations for both binding patterns. Using the procedure described in Materials and Methods, we selected from our previous simulations (control and such generated in the presence of FI10 or SK9) the two conformations with the lowest RMSD to either the Rod or the Twister segment. This procedure led to two model dimers bound at either the Rod binding site (E46-A56) or the Twister binding site (G68-A78). For a third dimer, we choose as chains the monomers with the highest strand content found in the earlier simulations. Sketches of the dimer models with and without viral protein fragments are shown in Figure 1. Two trajectories were followed over 500 ns for each of the three dimer systems, both in the presence of FI10 or SK9 and as a control in the absence of viral protein fragments. Note that after initial visual inspection of the time evolution of various quantities, we concluded that all simulations may need up to 400 ns for convergence (see also the discussion of Figure 2). Therefore, we use only the final 100 ns of each trajectory for the calculation of equilibrium quantities. Technical details on all simulated systems are listed in Table 1 and atomic coordinates of initial and final configurations are provided for each of the 18 trajectories as downloadable Supporting Information.

**Figure 1:**
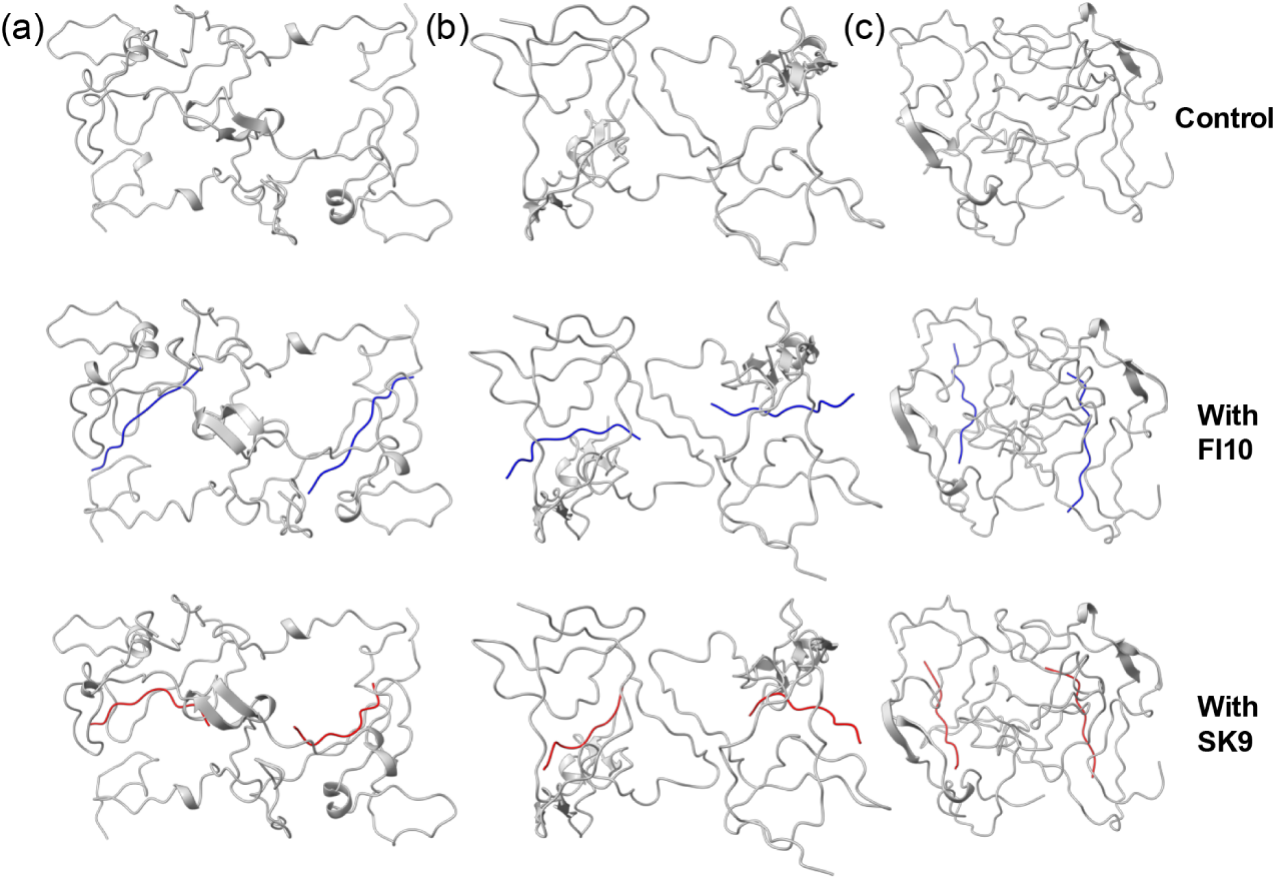
Sketches of the start configurations of our dimer simulations are shown, with the Rod Binding dimers shown in (a), Twister Binding dimers in (b), and the Beta Strand dimers in (c). The upper row shows the control, in the middle row is FI10 (in blue) binding to the dimers, and in the bottom SK9 (in red) binding to the dimers.

**Table 1:** Simulated Systems

**System Description**	**Atoms**	**Water Molecules**	**Independent Trajectories**	**Simulation Length (ns)**	**Total Sampling (ns)**
**Control Dimers**					
Rod Binding Dimer	125,105	40,275	2	500	1000
Twister Binding Dimer	129,288	41,666	2	500	1000
Beta Strand Dimer	73,476	23,096	2	500	1000
**Dimers with FI10**					
Rod Binding Dimer	119,579	38,317	2	500	1000
Twister Binding Dimer	128,570	41,308	2	500	1000
Beta Strand Dimer	72,692	22,716	2	500	1000
**Dimers with SK9**					
Rod Binding Dimer	125,735	40,375	2	500	1000
Twister Binding Dimer	127,386	40,924	2	500	1000
Beta Strand Dimer	72,717	22,735	2	500	1000

**Figure 2:**
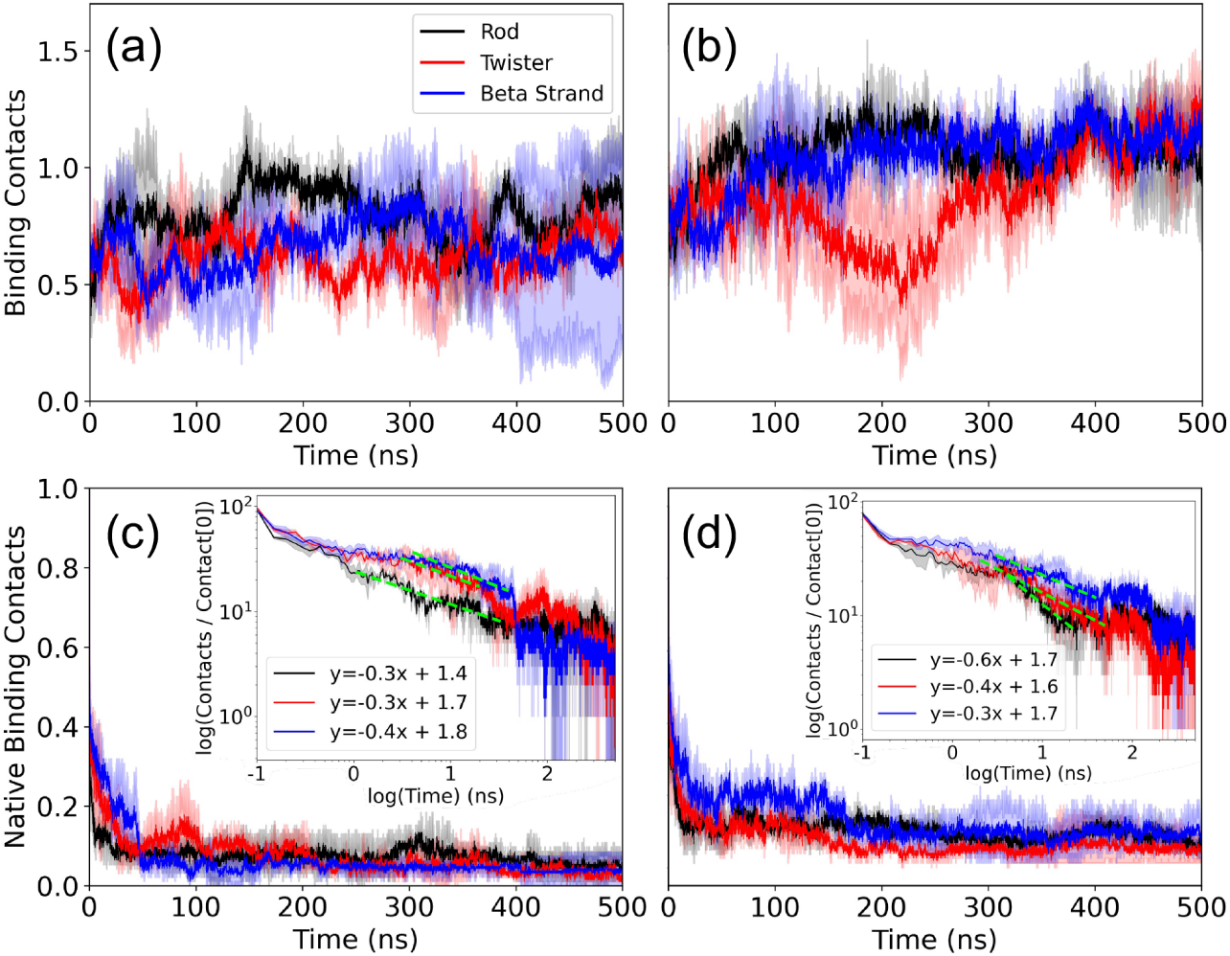
Time series of the number of contacts between the dimers and (a) FI10 and (b) the SK9 viral protein fragment are presented. Averages over two trajectories are shown, with the numbers normalized such that they are unity at the start (*t* = 0). In (c) and (d), we present the corresponding plots for the number of native contacts (i.e., contacts that are already observed at start), with the insets showing the time evolution of the same quantity in a log–log plot.

We start our analysis of these trajectories by examining the stability of the binding of FI10 and SK9 to the three dimer models. For this purpose, we show in Figure 2a the total number of contacts between the two FI10 segments and the respective αS dimer models as a function of time, and in Figure 2b the corresponding plots for SK9. For FI10, we observe for all three dimer conformations a decrease in the number of contacts, indicating a destabilization, with the loss being most pronounced for strand-dimers. We remark that the binding of both FI10 and SK9 is not concentrated to certain pockets but rather unspecific to the residues of the αS dimers, and contacts between the viral protein fragments and the dimers may form and decay. For instance, the number of contacts between SK9 and the twister dimer is similar between start and end of the trajectories but decreases in between by half before the SK9 later reattaches. Since the viral protein fragments can move over the dimer and even detach, we plot in addition in Figure 2c and d also the number of native contacts as a function of time, that is, the number of contacts that exist already at start. Here, we see in all cases a rapid decay, but with more native contacts surviving for SK9 binding to the dimers than for FI10 binding to the dimers. Initially, all models showed approximately 95 contacts between FI10 and the dimers. By the end of the simulation, only 5, 2, and 4 native contacts remained for the Rod Binding, Twister Binding, and Beta Strand dimers, respectively. In contrast, dimers with SK9 started with around 78 contacts, retaining 5, 4, and 6 native contacts for the Rod Binding, Twister Binding, and Beta Strand dimers at the end. The log–log plot in the insets demonstrates that the loss of native contacts can be described by a power law, which is indicative of the diffusive motion of the viral fragments over the dimer surface.

Combined, the four subfigures **2a–2d** indicate that SK9 binds more strongly to all three dimer models than FI10, with the difference being largest for the Beta Strand dimer and smallest for the Rod Binding dimer. Note that the curves in the four subplots have reached a plateau in the last 100 ns. While we cannot exclude the possibility that the systems are trapped in some metastable state, the similar behavior observed for the different quantities (and also in the individual trajectories) gives us confidence that our simulations have converged at this time. Hence, all our equilibrium properties rely on data taken over the last 100 ns of the trajectories. Specifically, we find that for this time interval, SK9 residues form about 5 contacts with αS chains in any of the three dimer models, while the numbers are with about 3 (Twister Binding) and 4 (Rod Binding and Beta Strand) smaller for FI10. Probabilities for SK9 or FI10 residues to form at least one contact with αS chains, evaluated over the last 100 ns and both trajectories, are given in Table 2 and again show a broad distribution, with stronger binding of SK9 residues to the dimers than seen for FI10, and binding of FI10 residues being especially weak to the Beta Strand dimer. Using the method of ref. 21, we find binding free energies for FI10 to be −96 kJ/mol (Rod Binding), −86 kJ/mol (Twister Binding), and −29 kJ/mol (Beta Strand). For SK9, the corresponding values are −127 kJ/mol (Rod Binding), −111 kJ/mol (Twister Binding), and −72 kJ/mol (Beta Strand). Hence, our data suggest that SK9 will affect the αS dimers more than FI10, and that the effect of SK9 is strongest for the Rod Binding dimer.

**Table 2:** Probabilities for SK9 or FI10 Residues to Form at Least One Contact with αS Chains, Evaluated over the Last 100 ns^a^

	**FI10**			**SK9**		
**Residue**	**Rod Binding Dimer**	**Twister Binding Dimer**	**Beta Strand Dimer**	**Rod Binding Dimer**	**Twister Binding Dimer**	**Beta Strand Dimer**
1	0.86	0.85	0.75	0.88	0.95	0.99
2	0.80	0.88	0.72	0.99	0.99	0.95
3	0.89	0.82	0.70	0.99	0.99	1
4	0.88	0.77	0.67	0.99	0.99	0.99
5	0.99	0.88	0.50	0.99	0.99	1
6	0.99	0.93	0.47	0.90	0.91	0.94
7	0.98	0.99	0.72	0.97	0.94	0.99
8	0.90	0.98	0.71	0.99	0.88	0.85
9	0.88	0.88	0.78	0.98	0.91	0.87
10	0.67	0.83	0.75	-	-	-

a Values are averaged over each fragment and both trajectories.

How does the binding of FI10 or SK9 alter the evolution of the dimers? The first quantity that we considered is the radius of gyration (*R*_g_), a measure of the dimer extension, and the average sheetness, i.e., the percentage of residues that were identified as strand-like. Averages of these and other quantities, taken over the final 100 ns and both trajectories, are shown for the system in Table 3 for Rod Binding, Twister Binding, and Beta Strand dimers. At start (*t* = 0), we find an *R*_g_ of about 2.8 nm and a sheetness of 6% for the Rod Binding dimer, 2.6 nm and 14% for Twister Binding dimers, and 2.2 nm and 20% for Beta Strand dimers. Averaged over the last 100 ns, the *R*_g_ values decrease for Twister Binding dimers by 0.2 nm in the control and 0.1 nm in the presence of FI10 and 0.5 nm in the presence of SK9, while the sheetness stays unchanged in all three cases. Similarly, the *R*_g_ values decrease for Rod Binding dimers by about 0.1 nm in the control and increase by 0.1 nm or stay unchanged in the presence of FI10 or SK9; however, the sheetness increases by 5% in the control while only by 1% in the presence of the viral protein fragments. The situation is different for the Beta Strand dimers, where the *R*_g_ increases by 0.1 nm for the control and 0.6 nm in the presence of FI10, but stays unchanged only in the presence of SK9. At the same time, the sheetness decreases here by 7% in all three cases. Hence, the presence of the viral fragments has little or no effect on the compactness or the sheetness of the dimer conformations, with only some minor stabilization seen for dimers with high strandness of the chains.

**Table 3:** Various Quantities Measured over the Last 100 ns in Simulations of Rod Binding, Twister Binding, and Beta Strand Dimers in the Presence of either FI10 or SK9, or in the Absence of the Viral Protein Fragments^a^

	**Control**		**With FI10**		**With SK9**	
**Rod Binding Dimer**	**Start Value**	**Avg last 100 ns**	**Start Value**	**Avg last 100 ns**	**Start Value**	**Avg last 100 ns**
Hydrophobic SASA (Å^2^)	15,114 (10)	11,564 (503)	15,202 (78)	12,188 (1275)	14,994 (11)	12,105 (1,175)
Hydrophilic SASA (Å^2^)	12,676 (62)	10,103 (244)	11,440 (52)	10,808 (430)	11,638 (64)	10,334 (845)
Total SASA (Å^2^)	27,790 (72)	21,667 (702)	26,641 (130)	22,966 (1705)	26,632 (74)	22,430 (2,019)
Interchain Contacts	45 (1)	155 (12)	50 (1)	43 (6)	48 (0)	87 (8)
Interchain Contacts with Fragment	-	-	94 (2)	74 (13)	80 (3)	85 (12)
Native Contacts	45 (1)	0 (0)	50 (1)	0 (0)	48 (0)	7 (6)
Native Contacts with Fragment	-	-	94 (2)	0 (0)	80 (3)	3 (1)
Distance Between Rod Binding Regions (Å)	6.1 (1)	11.6 (1.9)	6.2 (1)	30.7 (4.4)	6.1 (0)	10.8 (5.7)
Radius of Gyration (nm)	2.8 (0)	2.7 (0.7)	2.7 (0)	2.7 (5)	2.7 (0)	2.8 (8)
Percent Sheetness (%)	5.3 (0)	10.0 (0.5)	6.6 (0)	7.6 (4)	6.9 (0)	7.8 (3)

	**Control**		**With FI10**		**With SK9**	
**Twister Binding Dimer**	**Start Value**	**Avg last 100 ns**	**Start Value**	**Avg last 100 ns**	**Start Value**	**Avg last 100 ns**
Hydrophobic SASA (Å^2^)	13,236 (132)	11,573 (285)	13,761 (166)	12,026 (399)	13,555 (57)	11,094 (318)
Hydrophilic SASA (Å^2^)	10,949 (45)	10,300 (240)	10,043 (117)	10,697 (307)	10,138 (64)	9747 (291)
Total SASA (Å^2^)	24,185 (187)	21,603 (475)	23,803 (49)	22,723 (628)	23,692 (7)	20,842 (609)
Interchain Contacts	40 (2)	78 (41)	36 (4)	61 (16)	37 (5)	66 (68)
Interchain Contacts with Fragment	-	-	98 (0)	67 (6)	76 (1)	3 (1)
Native Contacts	40 (2)	5 (7)	36 (4)	2 (3)	37 (5)	4 (6)
Native Contacts with Fragment	-	-	98 (0)	3 (1)	76 (1)	9 (0)
Distance Between Twister Binding Regions (Å)	5.5 (0)	15.6 (12.8)	5.5 (1)	11.0 (1.1)	5.5 (1)	11.4 (8.4)
Radius of Gyration (nm)	2.6 (0)	2.4 (1)	2.6 (0)	2.5 (0.2)	2.5 (0)	3 (1)
Percent Sheetness (%)	13.6 (0)	14.9 (9)	13.8 (0)	13 (2)	13.8 (0)	16.8 (0)

	**Control**		**With FI10**		**With SK9**	
**Beta Strand Dimer**	**Start Value**	**Avg last 100 ns**	**Start Value**	**Avg last 100 ns**	**Start Value**	**Avg last 100 ns**
Hydrophobic SASA (Å^2^)	10,664 (1)	11,021 (430)	10,718 (122)	10,916 (815)	10,929 (139)	11,207 (223)
Hydrophilic SASA (Å^2^)	9293 (138)	9669 (262)	9198 (716)	9682 (852)	8614 (1)	9918 (245)
Total SASA (Å^2^)	19,956 (139)	20,670 (520)	19,416 (114)	20,598 (1666)	19,543 (136)	21,125 (405)
Interchain Contacts	110 (4)	104 (46)	107 (1)	104 (9)	100 (3)	90 (11)
Interchain Contacts with Fragment	-	-	93 (2)	41 (30)	78 (2)	88 (1)
Native Contacts	110 (4)	7 (1)	107 (1)	10 (4)	100 (3)	4 (1)
Native Contacts with Fragment	-	-	93 (2)	4 (5)	78 (2)	9 (5)
Radius of Gyration (nm)	2.2 (0)	2.3 (1)	2.2 (0)	2.8 (8)	2.2 (0)	2.2 (0)
Percent Sheetness (%)	20.0 (0)	13.2 (5)	21.4 (0)	13.0 (2)	22.1 (0)	15.4 (4)

a Averages over two trajectories for each system are shown, with standard deviations in parentheses.

We find a clearer signal in our measurements of the solvent-accessible surface area (SASA) in Figure 3 that describes the hydration of the dimers. For the Rod Binding dimer (Figure 3a–c) we observe in all three cases a decrease in SASA of about 4000 Å^2^ over the first 50 ns that continues for the control to a total loss of about 6000 Å^2^ at 500 ns, while a plateau is reached in the presence of SK9 (at a loss of about 4200 Å^2^) or FI10 (at a loss of about 3600 Å^2^). The difference in the control is especially visible for the SASA component resulting from hydrophilic residues. For these residues, 2500 Å^2^ are lost in the control but only 600 Å^2^ for FI10 and 1300 Å^2^ for SK9. The situation is different for Twister Binding dimers (Figure 3d–f) where the loss of SASA approaches a plateau after about 250 ns for the control (−3600 Å^2^) and SK9 (−2900 Å^2^), but increases again in the presence of FI10, resulting in a final loss of only 1100 Å^2^. This effect is again mostly due to contributions from hydrophilic residues, for which the SASA increases by about 650 Å^2^ in the presence of FI10 but decreases by about 380 Å^2^ in the presence of SK9 and 650 Å^2^ in the control. For Beta Strand dimers (Figure 3g–i) is again a plateau reached only after about 250 ns, with little change in SASA for the control (+760 Å^2^), and a larger gain in the presence of SK9 (+1600 Å^2^) or FI10 (+1200 Å^2^) resulting for SK9 mostly from hydrophilic residues (+1300 Å^2^). Hence, the viral protein fragments either minimize the loss of SASA for hydrophilic residues or even increase their exposure, with the effect stronger for FI10 than SK9 in Twister Binding and Rod Binding dimers.

**Figure 3:**
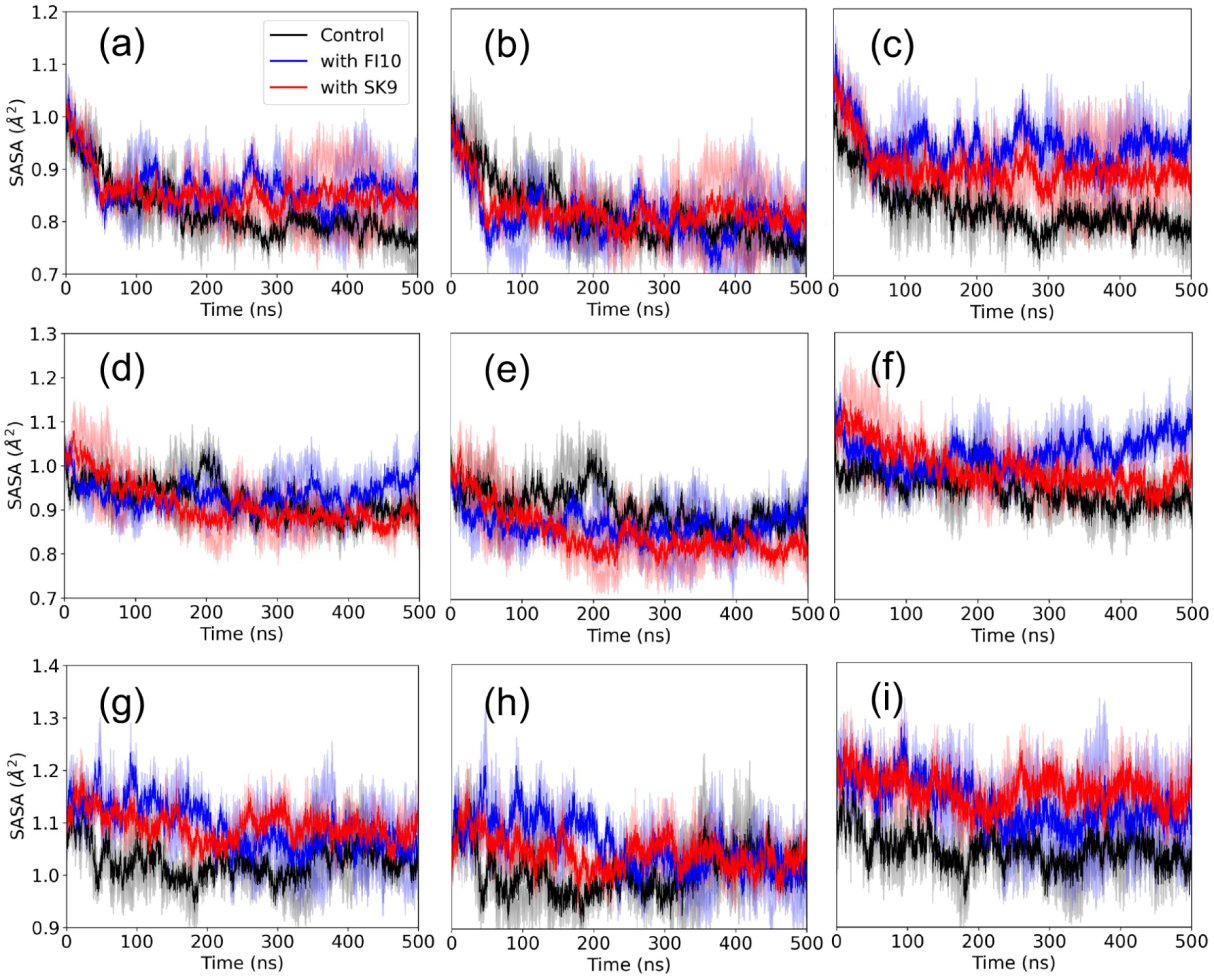
The solvent-accessible surface area (SASA) as a function of time for Rod Binding dimers (a-c), Twister Binding dimers (d-f), and Beta Strand dimers (g-i) for both control simulations and those in the presence of either FI10 or SK9. Averages over two independent trajectories are shown, with the values divided by the one at start. Values calculated over all residues are shown in the left row, while the center row shows the values calculated only for hydrophobic residues, and the right column shows the values calculated over hydrophilic residues.

The quantities discussed above describe global properties of the dimers. In order to understand in more detail how interaction with viral protein fragments affects the three dimer models, we continue our analysis by examining the number of interchain contacts between the two monomers in a dimer. The time evolution of this quantity, which is a measure for the stability of the dimer, is shown for the three systems in Figure 4. To allow for an easy comparison, we normalize our data again by dividing them by the respective start value. In all cases, we observe a quick plateauing for the simulations with FI10 or SK9 interacting with dimers, while (with the exception of the Beta Strand dimers) this process can take more than 200 ns for the control. For all three dimer models, we find that the number of such interchain contacts increases or stays similar to the start values (Figure 4a–c) while at the same time the number of native interchain contacts (i.e., contacts seen already at start, shown in Figure 4d–f) decreases rapidly. The increase in the total number of interchain contacts is smaller in the presence of SK9 or FI10 than in the control, see Table 3, with the effect stronger for FI10 than for SK9 and best seen for the Rod Binding dimer. On the other hand, the decrease in native contacts is similar in the control and FI10 for all three dimer models, but (with the exception of the Beta Strand dimer) lower for SK9. For instance, in the Rod Binding dimer, no native contacts are found in the final 100 ns of control simulations or such in the presence of FI10, but about 10 in such where SK9 is present. Hence, the presence of SK9 or FI10 stabilizes the original Rod Binding or Twister Binding dimer conformations, thereby delaying the decay of the respective starting conformations. This stabilization allows the two chains to move relative to each other and form additional contacts. The protecting effect seems to be stronger for SK9 in the Rod Binding dimer, while for the Twister Binding dimer, both viral protein fragments have a similar effect. No such stabilization is observed in Beta Strand dimers.

**Figure 4:**
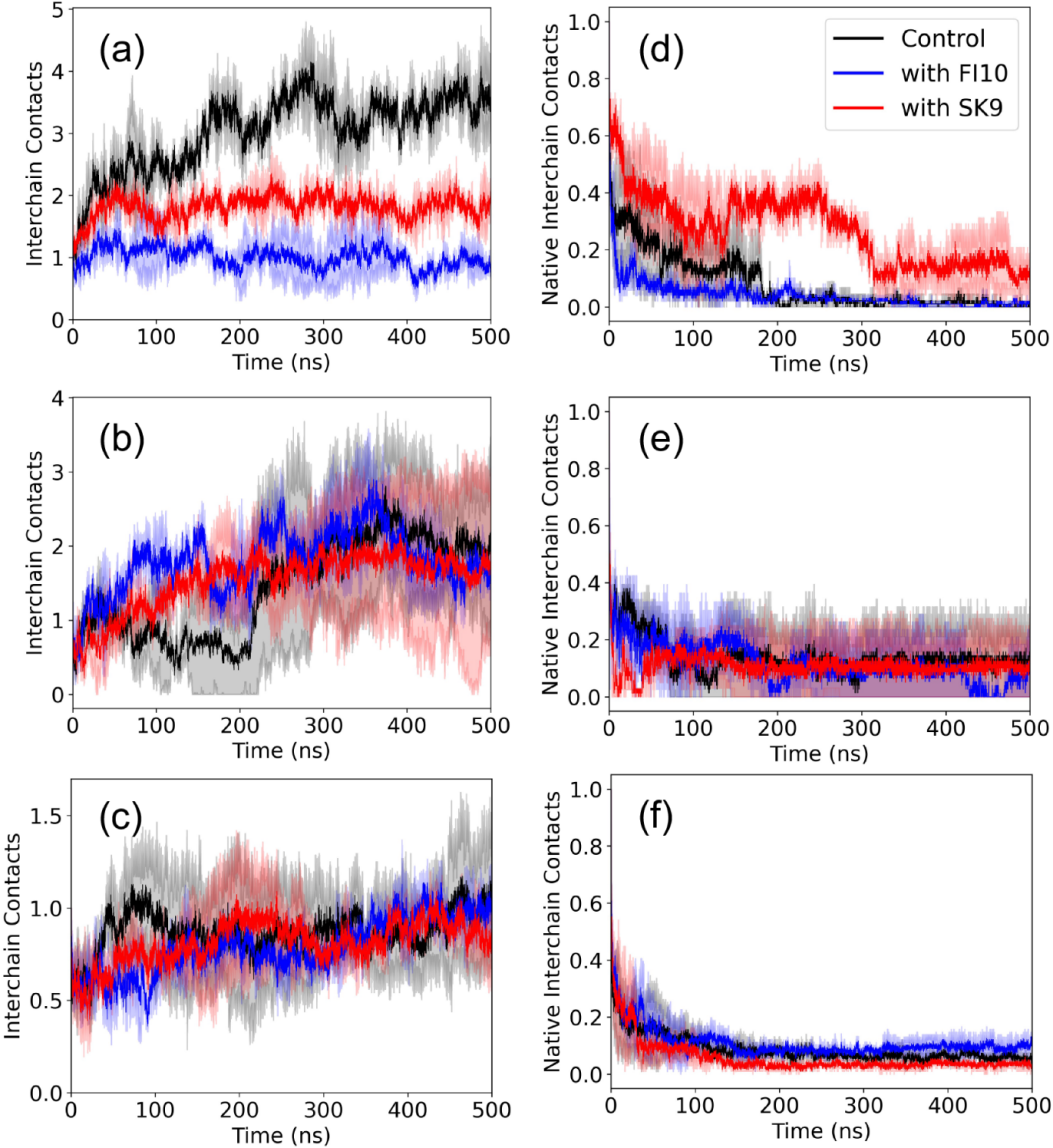
Number of interchain contacts (normalized to a start value of unity) between αS chains in the Rod Binding (upper row), Twister Binding (middle row), and Beta Strand (lower row) dimers is shown, both in the presence and absence of FI10 or SK9. Averages over two independent trajectories are presented. The plots in the right column show the corresponding numbers when considering only contacts that exist also at start.

The more pronounced effect of SK9 and FI10 on the Rod Binding and Twister Binding dimers, when compared with the Beta Strand dimers, may be because the Rod Binding and Twister Binding dimers have defined interaction interfaces. In order to test this hypothesis, we also look specifically into the interchain contacts at these interfaces (the segments of residues E46-A56 for the Rod Binding dimers and residues G68-A78 for Twister Binding dimers). We show in Figure 5a and b again the number of total contacts and the number of native contacts for Rod Binding dimers, and in Figure 5d and e for Twister Binding dimers, normalized to a start value of one for more easy comparison. Average values taken over the last 100 ns are given in Table 4.

**Table 4:** Interchain Contacts between the Binding Regions of Rod Binding or Twister Binding Dimers in the Presence or Absence of the Viral Protein Fragments SK9 and FI10

	**Rod Binding Dimers**						**Twister Binding Dimers**					
	**Control**		**With FI10**		**With SK9**		**Control**		**With FI10**		**With SK9**	
	**Start Value**	**Avg last 100 ns**	**Start Value**	**Avg last 100 ns**	**Start Value**	**Avg last 100 ns**	**Start Value**	**Avg last 100 ns**	**Start Value**	**Avg last 100 ns**	**Start Value**	**Avg last 100 ns**
**Interchain Contacts (Binding regions)**	23 (1)	2 (2)	26 (0)	0 (0)	24 (0)	9 (11)	24 (2)	8 (11)	22 (1)	3 (4)	22 (4)	10 (14)
**Native Contacts (Binding regions)**	23 (1)	0 (0)	26 (0)	0 (0)	24 (0)	5 (7)	24 (2)	3 (5)	22 (1)	2 (2)	22 (4)	4 (6)
**Distance Between Binding Regions (Å)**	6.1 (1)	11.6 (1.9)	6.2 (1)	30.7 (4.4)	6.1 (0)	10.8 (5.7)	5.5 (0)	15.6 (12.8)	5.5 (1)	11.0 (1.1)	5.5 (1)	11.4 (8.4)

**Figure 5:**
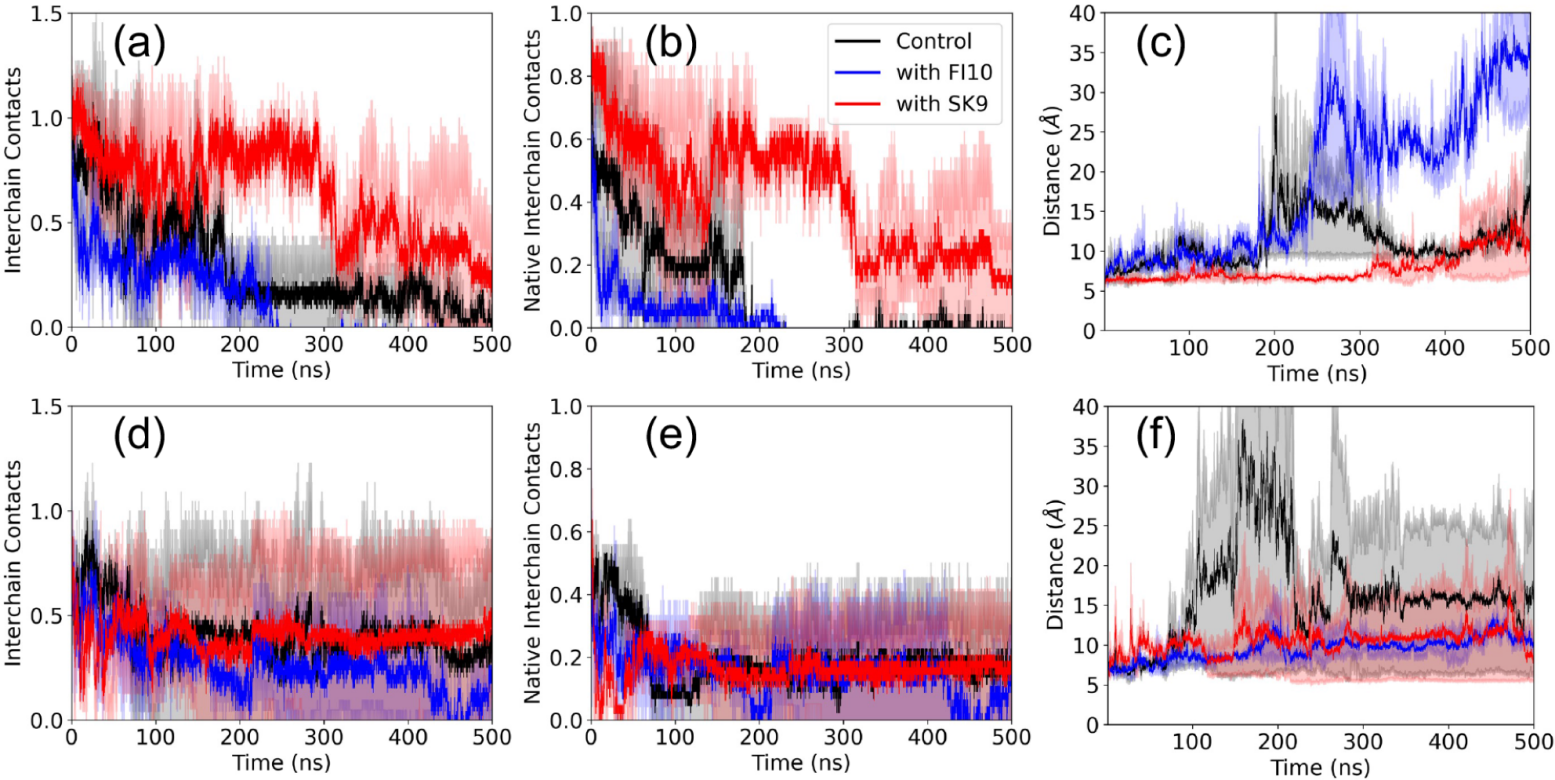
Number of interchain contacts (normalized to a start value of unity and averaged over two independent trajectories) between αS chains in the respective binding regions of Rod Binding dimers (upper row) and Twister Binding dimers (lower row) as a function of time for the control and in the presence of either FI10 or SK9. The right column shows the total number of such contacts, while the center column shows the number of native contacts, i.e., such already present at the start. The right column shows the distance between the two chains at the respective binding segment.

While the number of interchain contacts calculated over the full lengths of the chains increases in the two dimer models along the trajectories, this is not the case for the number of contacts between the two chains at the defined rod or twister binding interfaces. In both dimer models, these numbers decrease rapidly, and the differences between the number of such contacts and that of the native contacts, measured over the final 100 ns, is small; see Tables 2 and 4. This indicates that the dissolution of native contacts is often not compensated by the formation of new contacts, indicating a separation of the chains at the respective interface.

This is also seen in Figure 5c and f, where the distance between the two interfaces grows over time in all systems. For the Rod Binding dimers, we find that SK9 but not FI10 stabilizes binding at this interface. While in the control and in the presence of FI10, the distance between the two interfaces increases from the start and doubles its value at about 180 ns for the control, and at about 250 ns in the presence of FI10, it does not grow in the presence of SK9 for about 320 ns, and even later, the distance stays always lower than for the control and even more for FI10 where the Rod dimer appears to dissociate. Note that the time points where the distance between the rod interfaces starts increasing rapidly also corresponds with the times where rapid loss of interchain contacts is seen. On the other hand, in the Twister Binding dimer, the number of interchain contacts connecting the corresponding regions differs little between the control and trajectories where SK9 or FI10 are present, but the relative loss of contacts is smaller than for the Rod Binding dimer. However, the separation between the interfaces grows similar as for the Rod Binding dimer, with the growth being smaller in the presence of FI10 and SK9 than in the control. For instance, this distance reaches for the control at around 80 ns a value that is double that of its start values, and is settling over the last 100 ns at around 15 Å; while in the presence of SK9 and FI10, it takes about 180 ns before the initial distance has doubled, and afterward stays almost constant until reaching final values of about 10 Å. As for the Rod Binding dimer, the increase in distance between the binding interfaces is connected with the loss in native interchain contacts.

## 3 Conclusions

Using long molecular dynamics simulations, we studied the effect of two short SARS-COV-2 fragments, SK9 and FI10, on the stability of three αS-dimer models that we regard as potential seeds for the neurotoxic aggregates associated with the pathogenesis of Parkinson’s disease. We found that SK9 binds to all three models stronger than FI10 does and moves less over the surface of the dimers, even increasing the number of contacts with the dimers, especially the Beta Strand dimer. Both viral protein fragments have little effect on the time evolution of the number of interchain contacts in the Beta Strand dimer but, compared to the control, reduce the loosening and loss of secondary structure of the conformations and increase its solvent-accessible surface area, both for hydrophobic and hydrophilic residues. Hence, both fragments stabilize the Beta Strand dimer, but the effect is stronger for SK9, which also binds more strongly to this dimer model.

For Rod Binding and Twister Binding dimers, we do not observe any effect of the viral fragments on the secondary structure contacts, and even a larger loosening of the dimers that goes together with a smaller increase in the number of interchain contacts compared to the control. On the other hand, the decrease in native contacts is slightly less for FI10 and SK9 than in the control, with the effect being more pronounced for SK9. In the Rod Binding dimer, no native contacts are found in the final 100 ns of control simulations or such in the presence of FI10, but there are approximately 10 contacts when SK9 is present. This suggests that SK9, and to a smaller degree FI10, stabilizes the original conformation of the Rod Binding and Twister Binding dimers, delaying the movement of the two chains relative to each other, which could lead to forming additional contacts. The protective effect, especially of SK9, appears to be stronger for the Rod Binding dimer, where it results in a reduced decrease in solvent-accessible surface area, which is influenced by both hydrophobic and hydrophilic residues. Note that for the Twister Binding dimer, the decrease in SASA observed in the control is reversed for FI10 but not for SK9. This reversal is due to a strong increase in the SASA for hydrophilic residues. An increase (or smaller decrease) in the exposure of hydrophilic residues in the presence of FI10 (and to a lesser extent also of SK9) is observed in both dimer models and seems to be one of the mechanisms for the effect of the protein fragments on the dimers. Rod Binding and Twister Binding dimers were designed to have binding interfaces at the segments observed in two experimentally resolved fibril polymorphs. Measuring the number of interchain contacts and the distance between the respective segments, we find that the presence of SK9 or FI10 stabilizes both the twister and rod interface, but the effect is more pronounced for the Rod Binding dimer. In this case, we observe a strong protective effect by SK9 but none by FI10, whereas in the control, the chains even separate.

In summary, our results indicate that the two viral protein fragments may stabilize the αS-dimer, potentially allowing them to seed neurotoxic aggregates. The extent and mechanism depend both on the fragment and the dimer model, but our results suggest that SK9 has a larger effect than FI10, stabilizing existing structural elements (such as binding interfaces or secondary structures) and encouraging exposure of hydrophilic residues. Especially, we find that SK9 seems to strongly stabilize Rod Binding dimers. This allows them to persist longer in solution, increasing the likelihood that early aggregation species adopt rod-like conformations. Since protofibrils can serve as nucleation seeds for elongation into mature fibrils, an increase in dimers with stabilized rod interfaces over twister interfaces can shift the population distribution toward rod-like fibril formation. Preferential stabilization of rod-binding dimers by SARS-CoV-2 protein fragments (especially SK9) can therefore enable fibril nucleation to proceed via a pathway that favors rod-like fibrils over alternative polymorphs. Hence, this differential stabilization suggests a mechanism by which viral proteins can modulate amyloid formation of αS, and, as the cell toxicity of the various fibril polymorphs differs, potentially also the severity and pathogenesis of Parkinson’s Disease.

## 4 Materials and Methods

### 4.1 System Preparation

Our study aims to understand αS aggregation by investigating αS dimers as being the simplest oligomers. We use all-atom molecular dynamics (MD) simulations to study the change in the conformational ensemble of αS dimers induced by the presence of the ten-residue segment _194_FKNIDGYFKI_203_ (FI10) of the spike protein and the nine-residue segment _54_SFYVYSRVK_62_ (SK9) of the envelope protein from SARS-COV-2. The initial configurations for dimer models were obtained by docking homogeneous monomers taken from previous MD simulations of the αS monomer.^7,8^ The equilibrated monomer conformations were selected for the Rod Binding and Twister Binding dimers based on the structural similarity of the protofilament interfaces to the same regions in cryo-EM structures of the corresponding Rod and Twister αS fibrils, as measured by root-mean-square deviation (RMSD). Specifically, the selection was based on the protofibril binding region (E46-A56) in the Rod polymorph (PDB ID: 6CU7) and (G68-A78) in the Twister polymorph (PDB ID: 6CU8).^9^ For the Beta Strand dimer, monomers were chosen from conformations with the highest percentage of sheetness as determined by VMD and the STRIDE algorithm.^10^ Dimers were generated by docking identical monomers by HADDOCK program using standard protein–protein parameters.^11,12^ For the Rod and Twister Binding dimers, the protofibril binding regions were selectively docked to form dimers with these regions in contact. In all models, we capped the N- and C-terminal of each αS chain with an NH_3_^+^ and a COO^–^ group.

Simulations starting from the αS dimer models described above serve as controls for simulations in which FI10 or SK9 peptides are also present. Initial configurations of FI10 and SK9 were prepared as described in earlier work,^7,8^ with the N- and C-terminal of FI10 capped by an NH_3_^+^ and −COO^–^ group, respectively, and, in order to stay consistent with earlier work, SK9 capped by an NH_3_^+^ and −CONH_2_ group. To produce the initial conformations for our simulations, we docked two FI10 and SK9 segments to each dimer at binding sites predicted by HADDOCK when using standard protein-peptide parameters.^11,12^ A single fragment was docked to each monomer in the dimer, producing symmetrical initial configurations. Note that the viral protein segments are not fixed but can move freely throughout the simulations and may detach from the αS chains. The so obtained start configurations are shown in Figure 1, and their atomic coordinates are provided as downloadable Supporting Information.

### 4.2 General Simulation Protocol

The dimer systems were simulated with the GROMACS 2022 package^13^ using the CHARMM36m all-atom force field^14^ with TIP3P explicit water.^15^ Hydrogen atoms were added with the *pdb* 2gmx module of the GROMACS suite.^13^ The start configurations for each system were placed at the center of a cubic box with periodic boundary conditions and an edge length in each direction of 10.56 nm for the Rod Binding dimer, 10.81 nm for the Twister Binding dimer, and 10.74 nm for the Beta Strand dimer. The simulation boxes were solvated with water molecules, and Na^+^ and Cl^–^ ions were added at a physiological ion concentration of 150 mM NaCl to neutralize the system. Table 1 lists the total number of atoms and the number of water molecules in each system. Energy minimization occurred by steepest decent for up to 50,000 steps, followed by a short molecular dynamics simulation at 310 K for 200 ps at constant volume and an additional 200 ps at constant pressure (1 atm), constraining the positions of heavy atoms with a force constant of 1000 kJ mol^–1^ nm^–2^.

During the trajectories, the temperature was held constant at 310 K by a v-rescale thermostat^16^ with a coupling constant of 0.1 ps, and the pressure was maintained at a constant 1 atm by using the Parrinello–Rahman barostat^17^ with a pressure relaxation time of 2 ps. The SETTLE algorithm^18^ keeps water molecules rigid, and protein bonds involving hydrogen atoms are restrained to their equilibrium length with the LINCS algorithm.^19^ We used a time step of 2 fs for integrating the equations of motion. Due to periodic boundary conditions, we used the particle-mesh Ewald (PME) technique to calculate the long-range electrostatic interactions, with a real-space cutoff of 12 Å and a Fourier grid spacing of 1.6 Å. Short-range van der Waals interactions were truncated at 12 Å, with smoothing starting at 10.5 Å. In this study, we considered two trajectories of 500 ns for each model with different initial velocity distributions.

### 4.3 Trajectory Analysis

GROMACS tools^13^ and VMD are used to analyze trajectories. VMD is used to visualize conformations.^20^ GROMACS tools are used to calculate root-mean-square deviation (RMSD), radius of gyration (Rg), solvent-accessible surface area (SASA), distance between binding regions, and contact frequencies. The calculation of SASA relied on a spherical probe with a radius of 1.4 Å. Contacts are defined by a cutoff of 4.5 Å in the closest distance between heavy atoms in a residue pair. Residue-wise secondary structure propensity is calculated using VMD and the STRIDE algorithm.^10^ Helicity and sheetness are defined as the percentage of residues with an α-helical (i.e., assigned an “H” by STRIDE) or beta-strand (an “E” for extended configuration or a “B” for an isolated bridge) secondary structure in every frame of the trajectory. Binding free energies are calculated using the method described in ref.^21^ where one must account for the difference in chain lengths when calculating the concentration, *C*_*sim*_.

## Supplementary Material



## Data Availability

The data that support the findings of this study are available in the Supporting Information and are publicly accessible at https://github.com/ouhansmannlab/alpha_synuclein.git

## Notes

Start and final configurations of all trajectories in PDB format (ZIP)
